# Molecular Cluster‐Controlled Quasi‐Epitaxial CZTSSe/CdS Heterojunction Enables 12.3% Efficiency of Flexible Solar Cells

**DOI:** 10.1002/advs.202520208

**Published:** 2025-12-08

**Authors:** Weihao Xie, Yifan Li, Quanzhen Sun, Weihuang Wang, Renjie Wang, Caixia Zhang, Jionghua Wu, Hui Deng, Shuying Cheng

**Affiliations:** ^1^ College of Physics and Information Engineering Institute of Micro‐Nano Devices and Solar Cells Fuzhou University Fuzhou 350108 P. R. China; ^2^ Jiangsu Collaborative Innovation Center of Photovoltaic Science and Engineering Changzhou 213164 P. R. China

**Keywords:** CdS deposition, CZTSSe, epitaxial heterojunction, flexible solar cell

## Abstract

Flexible Cu_2_ZnSn(S,Se)_4_ (CZTSSe) solar cells have garnered significant attention in photovoltaics. Interface defects in CZTSSe/CdS heterojunctions drive carrier recombination, leading to substantial open‐circuit voltage (*V_OC_*) loss. Herein, a deposition strategy is proposed to achieve quasi‐epitaxial heterojunctions by controlling the CdS molecular clusters during the chemical bath deposition (CBD) process. The nucleation rate and size of clusters are regulated by manipulating the stirring process and a sealed NH_3_ atmosphere. Small‐sized CdS molecular clusters, densely adsorbed onto the CZTSSe surface at a controlled low rate, pair with dangling bonds to form the quasi‐epitaxial heterojunction structure. This effectively suppresses interface defects and mitigates tunnel‐enhanced recombination, resulting in an increased *V_OC_* of 503 mV. The epitaxial growth of CdS thin films facilitates the formation of ultrathin buffer layers, thereby enhancing the short‐wavelength transmittance of the window layer, resulting in a 15% boost in short‐circuit current density (*J_SC_*). Finally, the flexible CZTSSe solar cell achieves a power conversion efficiency (*PCE*) of 12.3% and demonstrates exceptional mechanical stability, retaining over 95% of its initial efficiency after thousands of bending cycles. The developed quasi‐epitaxial heterojunction strategy suppresses interfacial recombination, offering a promising route toward high‐efficiency flexible kesterite solar cells.

## Graphical abstract

By controlling the synthesis of CdS molecular clusters, a quasi‐epitaxial heterojunction is directly constructed during chemical bath deposition, enabling flexible kesterite solar cells to achieve an efficiency of 12.3%.

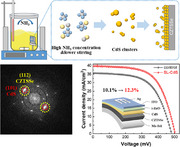

## 1 Introduction

Cu_2_ZnSn(S,Se)_4_ (CZTSSe) has garnered wide attention in photovoltaics due to its high absorption coefficient, abundant elemental compositions, tunable bandgaps, non‐toxic properties, and flexible ability.^[^ ^1^, ^2^, ^3^ ^]^ Flexible CZTSSe thin‐film solar cells have demonstrated commercial potential in indoor photovoltaics, building‐integrated photovoltaics, and wearable devices.^[^ ^4^, ^5^, ^6^ ^]^ Nowadays, flexible CZTSSe solar cells are developing rapidly, with a power conversion efficiency now exceeding 13%.^[^ ^7^, ^8^ ^]^ However, there is still a significant gap from the Shockley–Queisser limit^[^ ^9^, ^10^ ^]^ due to severe non‐radiative recombination in CZTSSe devices.^[^ ^11^, ^12^ ^]^ In recent years, through precursor optimization,^[^ ^13^, ^14^, ^15^, ^16^ ^]^ cation doping,^[^ ^3^, ^17^, ^18^, ^19^, ^20^, ^21^ ^]^ and selenization optimization,^[^ ^22^, ^23^, ^24^ ^]^ the deep‐level defects and non‐radiative recombination have been effectively suppressed to optimize the crystallinity of CZTSSe film. Meanwhile, the interface of the CZTSSe/CdS heterojunction also plays a decisive role in the performance of CZTSSe solar cells. High‐density interface defects lead to severe carrier recombination, causing open‐circuit voltage (*V_OC_*) loss.^[^ ^12^, ^25^ ^]^ In flexible devices, heterojunction quality also severely affects bending tolerance.^[^ ^4^, ^26^, ^27^ ^]^ Therefore, optimizing the CZTSSe/CdS heterojunction is one of the key points for improving flexible CZTSSe solar cells.

Recently, researchers focused on the optimization strategies for the CdS buffer layer to improve the heterojunction quality of the CZTSSe solar cells. Metal cation doping in CdS film can adjust the bandgap to achieve energy level matching in heterojunctions,^[^ ^9^, ^28^, ^29^ ^]^ and oxygen‐containing post‐treatment can suppress deep‐level S vacancy defects in CdS.^[^ ^30^, ^31^ ^]^ Post‐treatment of the CZTSSe thin film surface by a modification layer can passivate deep‐level defects on the absorber surface, thereby suppressing carrier recombination at the heterojunction interface.^[^ ^32^, ^33^, ^34^ ^]^ The above methods effectively optimized the carrier transport properties of the heterojunction. Meanwhile, the fundamental issue facing the CZTSSe/CdS heterojunction is the high density of interface defects caused by interface mismatch, which greatly contributes to *V_OC_* loss through tunnel‐enhanced interface recombination. Constructing an epitaxial heterojunction interface serves as an effective strategy to mitigate interface mismatch issues.^[^ ^35^, ^36^, ^37^, ^38^ ^]^ Liu et al. annealed CZTS films with a mixture of sulfur and SnS vapor, resulting in the enhancement of Cd^2^⁺ diffusion during the deposition process, enabling CdS epitaxial growth.^[^ ^39^ ^]^ Gong et al. subjected CZTSSe/CdS heterojunctions to post‐annealing treatment, forming the re‐arrangement of cations at the interface, thereby achieving heterojunctions with an epitaxial structure.^[^ ^40^ ^]^ For flexible CZTSSe devices, the direct construction of epitaxial interfaces through CBD can effectively reduce manufacturing costs and suppress interface recombination. In addition, the high‐quality CdS layer and interface are also beneficial in preventing the efficiency degradation after bending.

The CdS buffer layers in CZTSSe devices are commonly prepared by the chemical bath deposition (CBD) method.^[^ ^41^ ^]^ Under the combined effects of heating and stirring during the CBD process, Cd^2+^ is gradually released from [Cd(NH_3_)_4_]OH_2_ and reacts with S^2^^−^ (from thiourea) to form CdS clusters.^[^ ^42^ ^]^ When the CdS cluster size is large, the dangling bonds on the CZTSSe surface cannot be completely paired, resulting in severe interface mismatch between the CdS and CZTSSe films.^[^ ^43^ ^]^ Moreover, the pores within CdS aggregates obstruct carrier transport and propagate under bending stress, leading to performance degradation in flexible devices. By controlling the NH_3_ concentration and stirring rate to inhibit the reaction rate between Cd^2+^ and S^2−^, the formation rate and size of CdS clusters can be reduced. During this process, tiny CdS nanocrystals are tightly and orderly adsorbed onto the CZTSSe surface and paired with the dangling bonds. Therefore, decreasing the size of CdS clusters during the CBD process is conducive to directly achieve a quasi‐epitaxial interface on the CZTSSe surface.

In this work, the quasi‐epitaxial CZTSSe/CdS heterojunctions are constructed to suppress interface recombination and *V_OC_* loss for high‐performance flexible CZTSSe solar cells. The formation of CdS molecular clusters is controlled by the optimal ammonia‐sealed method in the CBD process. The CdS nanocrystals at the interface exhibit a highly oriented and densely packed arrangement, achieving a quasi‐epitaxial CZTSSe/CdS heterojunction. The defect passivation and reduced tunnel‐enhanced interface recombination are characterized by low‐temperature measurements. The high‐quality CdS deposition process facilitated the fabrication of a thinner buffer layer, thereby enhancing the window layer transmittance and the device bending tolerance. Ultimately, the flexible CZTSSe solar cell achieves a power conversion efficiency of 12.3%, benefiting from suppressed photocarrier recombination at the high‐quality heterojunction. Remarkably, the device maintains stable performance even after thousands of bending cycles.

## 2 Results and Discussion

CdS thin films employed as buffer layers in flexible CZTSSe thin‐film solar cells are typically prepared using CBD methods. To ensure controlled reaction kinetics and prevent premature precipitation of the target compound, pre‐forming metal complexes effectively restricts the concentration of free metal ions in the reaction bath. Ammonium hydroxide is used as a coordinating agent in the CBD process for CdS thin films. The general chemical reaction is as follows:

(1) $$\mathrm{CdS}O_{4}+2NH_{4}\mathrm{OH}⇌\mathrm{Cd}{\left(\mathrm{OH}\right)}_{2}+{\left(NH_{4}\right)}_{2}SO_{4}$$

(2) $$\mathrm{Cd}{\left(\mathrm{OH}\right)}_{2}+4NH_{4}\mathrm{OH}⇌{\left[\mathrm{Cd}{\left(NH_{3}\right)}_{4}\right]}^{2+}+2OH^{-}+4H_{2}O$$

(3) $${\left(NH_{2}\right)}_{2}\mathrm{CS}+2OH^{-}\to CH_{2}N_{2}+2H_{2}O+S^{2-}$$

(4) $${\left[\mathrm{Cd}{\left(NH_{3}\right)}_{4}\right]}^{2+}+S^{2-}\to \mathrm{CdS}+4NH_{3}$$

**Figure 1:**
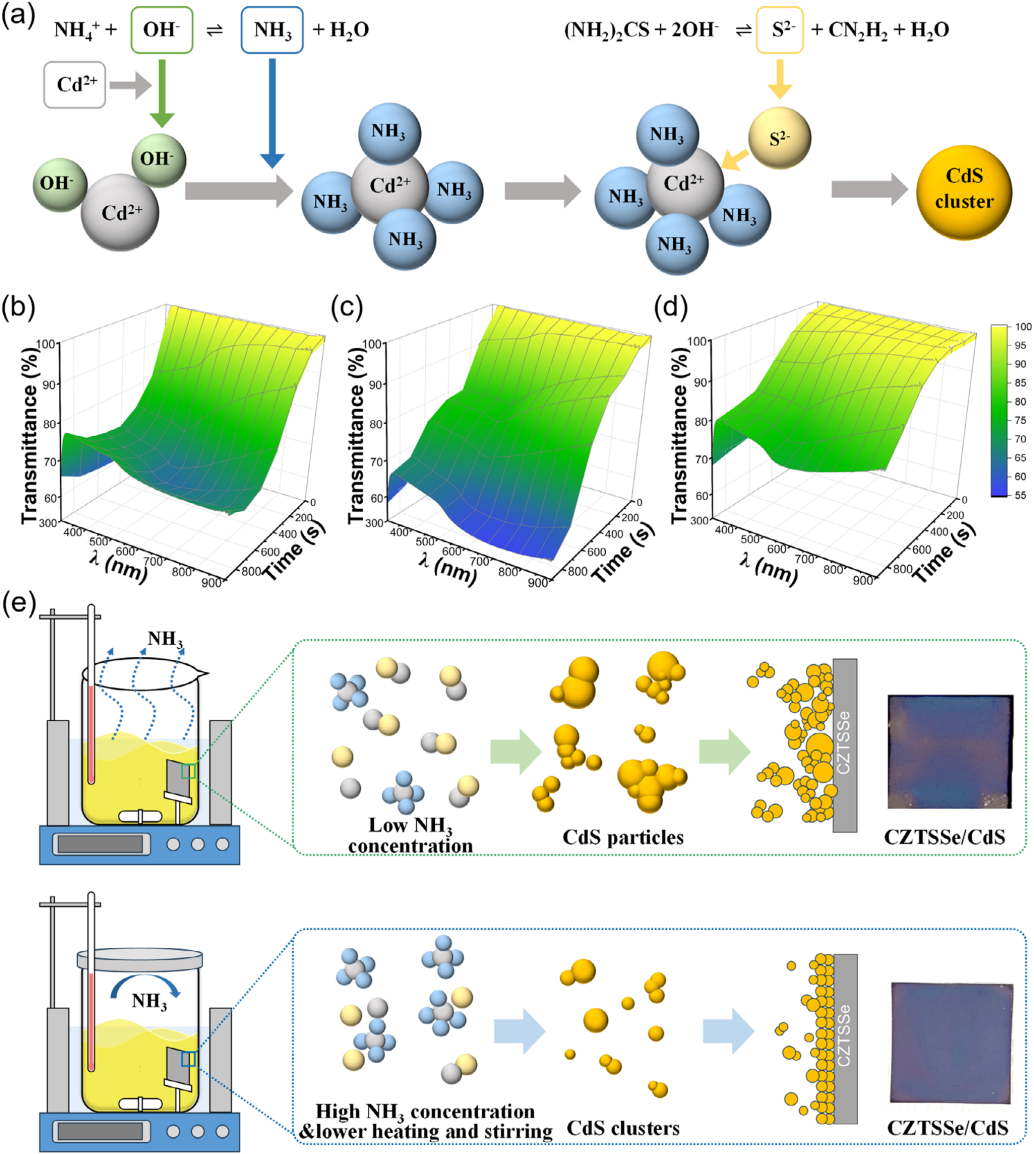
a) The schematic diagram of the formation process of CdS clusters. b–d) Time‐resolved transmittance of CdS CBD solutions for control method (b), S‐CdS method (c), and SL‐CdS method (d). e) Schematic diagrams of the CdS CBD process.

The schematic diagram of the formation process of CdS clusters is shown in **Figure** **1**a. The NH_3_ concentration can prevent the precipitation of hydroxides and coordinate with Cd^2+^. The Cd(OH)_2_ formed by the reaction between Cd^2+^ and OH^−^ complexes with NH_3_ to form [Cd(NH_3_)_4_]^2+^. In an alkaline environment, thiourea decomposes to release S^2−^, which adsorbs onto [Cd(NH_3_)_4_]^2+^. CdS molecular clusters are formed from the complex after NH_3_ dissociates. During the CBD process, the heating and stirring can promote NH_3_ volatilization and increase the molecular kinetic energy of the reaction, leading to the direct decomposition of [Cd(NH_3_)_4_]^2+^ ions and the release of free Cd^2+^ ions. Consequently, precise control of NH_3_ loss is critical for modulating the growth kinetics of CdS thin films. To address this motivation, we propose three CBD methods for comparative analysis of the deposition processes and their impacts on device performance. The control group employs the conventional CdS CBD method using an open reaction vessel. The sealed‐atmosphere‐based CBD method is used to reduce NH_3_ loss(named as S‐CdS). And the method further reducing molecular kinetic energy by lowering heating temperatures and late‐half stirring is named as “SL‐CdS.” Detailed process parameters are provided in the Experimental Section.

Through the time‐resolved transmittance measurements of the CBD solution, we analyzed the differences in the growth of CdS clusters for the three kinds of CBD processes. In the control group, the solution's transmittance is rapidly decreased and then recovered in the later stages (see Figure 1b). The speedy decrease in transmittance throughout the entire test wavelength range indicates that alongside the exceedingly rapid formation rate of CdS (absorption range of 500–560 nm) and the sizes of the CdS clusters are large enough to severe light obstruction and scattering effects. The subsequent recovery of transmittance in the late CBD stage further indicates that Cd^2^⁺ depletion and CdS particles precipitation in the reaction vessel. Compared to the control method, the solution transmittance of the S‐CdS CBD decreases more linearly and does not rebound in the later stages (see Figure 1c). The slower decrease of transmittance in the early stages of the process is evidence of decreased CdS formation rate. The persistent decline in transmittance in the later stages suggests that Cd^2+^ ions remain unexhausted, sustaining the reaction and leading to continuous accumulation of CdS nanoparticles. However, in the late stage of CBD, the persistently low transmittance in the 600–900 nm range implies excessive large CdS particles formation compromising film quality. The SL‐CdS method effectively addresses this limitation by suppressing molecular kinetic energy. As shown in Figure 1d, this strategy moderates the transmittance declines dynamics. Crucially, the pronounced transmittance increase in the 600–900 nm range confirms successful inhibition of large‐particle formation. These observations underscore the critical influence of NH_3_ atmosphere regulation and process parameter optimization on CdS film quality.

The prepared CdS thin films by the three methods are characterized by space charge limitation currents (SCLC) and fluorescence luminescence (FL) spectra. As shown in Figure S1 (Supporting Information), the SCLC curve of the SL‐CdS sample has the lowest trap filling limit voltage (*V_TFL_*), which is related to the lowest bulk defect density. The test samples used for FL characterization were prepared on ITO substrates. The spectra shows that SL‐CdS has the lowest excitation peak intensity (see Figure S2, Supporting Information), which is due to enhanced quenching ability resulting from better charge transport capability. The significant performance differences among CdS films originate from the sizes of CdS molecular clusters and the deposition mechanisms during the CBD processes. Figure 1e shows the schematic diagrams of the CdS thin films deposition processes and corresponding sample photographs. The [Cd(NH_3_)_4_]^2+^ complex is unstable and decomposes upon heating and stirring to liberate abundant Cd^2+^ ions. These ions rapidly nucleate into CdS clusters of polydisperse sizes, ultimately forming a porous CdS film. Concurrently, the heterogeneous orientation of large CdS clusters adsorbed on the CZTSSe surface induces nonuniform adsorption kinetics for subsequent clusters, thereby compromising film homogeneity. The SL‐CdS strategy synergistically reduces both the stability of [Cd(NH_3_)_4_]^2+^ complex and molecular kinetic energy, effectively altering the formation and deposition mechanism of CdS molecular clusters. This dual modulation achieves precise control over cluster formation kinetics while maintaining monodisperse nanoparticle sizes throughout the CBD process. Consequently, CdS molecular clusters are uniformly adsorbed on the CZTSSe surface. The resulting high‐quality CdS thin film is the key to constructing an optimized heterojunction interface, effectively suppressing carrier recombination.

**Figure 2:**
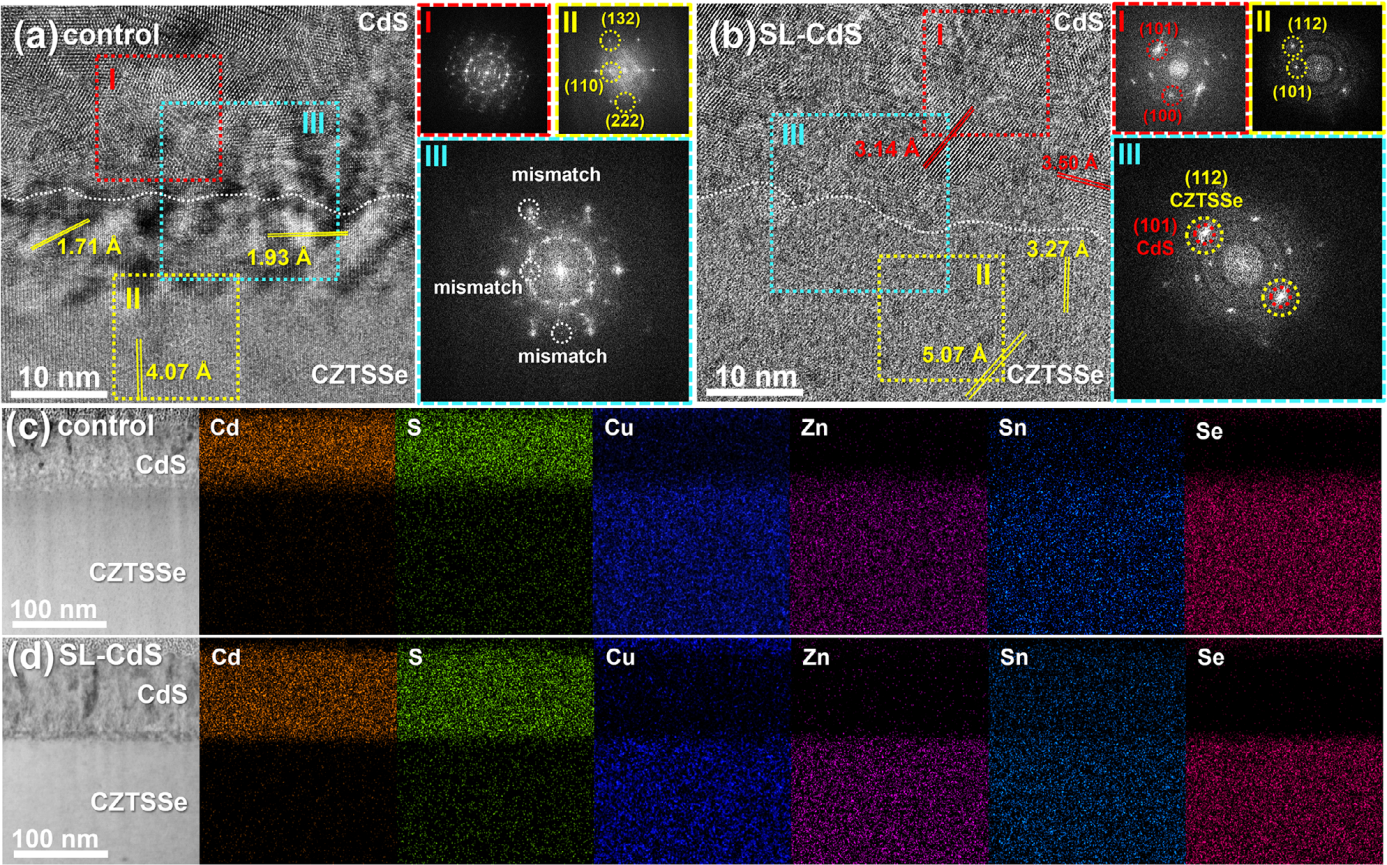
a,b) High‐resolution TEM images of the CZTSSe/CdS heterojunction interface, with FFT spectra taken from CdS, CZTSSe, and interface regions of the control sample (a) and SL‐CdS sample (b). c,d) TEM‐EDS mapping images of CZTSSe/CdS cross section of control sample (c) and SL‐CdS sample (d).

High‐resolution transmission electron microscopy (HRTEM) is used to analyze the cross‐section of the CZTSSe/CdS interface. **Figure** **2**a presents a cross‐sectional HRTEM image of the control sample heterojunction, alongside the corresponding fast Fourier transform (FFT) patterns acquired from the CdS layer, the CZTSSe absorber, and the interface region. The FFT spectra of CdS consist of multiple overlapping diffraction spots and diffraction rings, revealing that the CdS film prepared by the control method is composed of numerous CdS particles with different crystal faces. The CZTSSe region exhibits clear diffraction spots, with interplanar spacings of 4.07, 1.71, and 1.93 Å for the (110), (132), and (222) crystal orientations, respectively. From the FFT spectra of the interface, it is observed that the diffraction spots of CdS do not overlap with those of CZTSSe, indicating interfacial lattice mismatch. In contrast, the SL‐CdS sample (Figure 2b) displays a marked different interfacial structure. The FFT pattern of CdS reveals well‐defined diffraction spots, with interplanar spacings of 3.14 and 3.50 Å for the (101) and (100) crystal planes, respectively. The FFT spectra of the CZTSSe region exhibit (112) and (101) crystal orientations, with interplanar spacings of 3.27 and 5.01 Å, respectively. The diffraction image from the interface region shows an overlapping reflection between CdS (at (101)) and CZTSSe (at (112)), indicating that the hexagonal CdS prepared by the SL‐CdS method epitaxially grows at the interface.^[^ ^35^, ^39^ ^]^ TEM‐EDS mapping is conducted to analyze the cross‐sectional morphology and elemental distribution of CZTSSe/CdS. Figure 2c shows that the CdS film layer of the control sample contains a large number of CdS particles with diffused element signals at the interface. Cu and Zn show relatively obvious diffusions at the interface, indicating the existence of Cu_x_S and ZnS clusters, which are harmful to carrier transport. The large CdS particles accumulating on the CZTSSe surface cause misorientation and loose interfaces. As shown in Figure 2d, the CdS film of the SL‐CdS sample is uniform and compact with no particles and less element diffusion at the interface. The suppression of cation diffusion may originate from the early adsorption of dense CdS clusters in the CBD process. This significantly reduces the dissolution of Cu and Zn, minimizes the formation of harmful subphases at the interface, and ensures an interface with low defect density and high transport capability. Figure S3 (Supporting Information) presents the SEM surface and cross‐sectional morphologies of the heterojunction samples. The CdS film fabricated by the control method on the CZTSSe layer shows a porous surface and a cross‐section characterized by extensive agglomerate accumulation and interfacial voids. In contrast, the CdS film prepared via the SL‐CdS method exhibits a dense surface and a cross‐section of vertically aligned, densely packed grains. No alteration is observed in the position or half‐width of the CZTSSe (112) diffraction peak following CdS deposition, as evidenced by XRD (Figure S4, Supporting Information). This demonstrates that the crystalline quality and lattice structure of the CZTSSe absorber remain intact during the buffer layer growth process. In conjunction with the preceding discussion, the high NH_3_ atmosphere reduces the decomposition of [Cd(NH_3_)_4_]^2+^ complexes, thereby suppressing the release rate of Cd^2+^. Reduced stirring and heating not only inhibit NH_3_ volatilization but also decrease the molecular kinetic energy in the solution. Under the combined effects of these conditions, the size and deposition rate of CdS molecular clusters during the early stages of the CBD process are strictly controlled. These clusters adsorb tightly onto the CZTSSe surface, forming a quasi‐epitaxial heterojunction.

**Figure 3:**
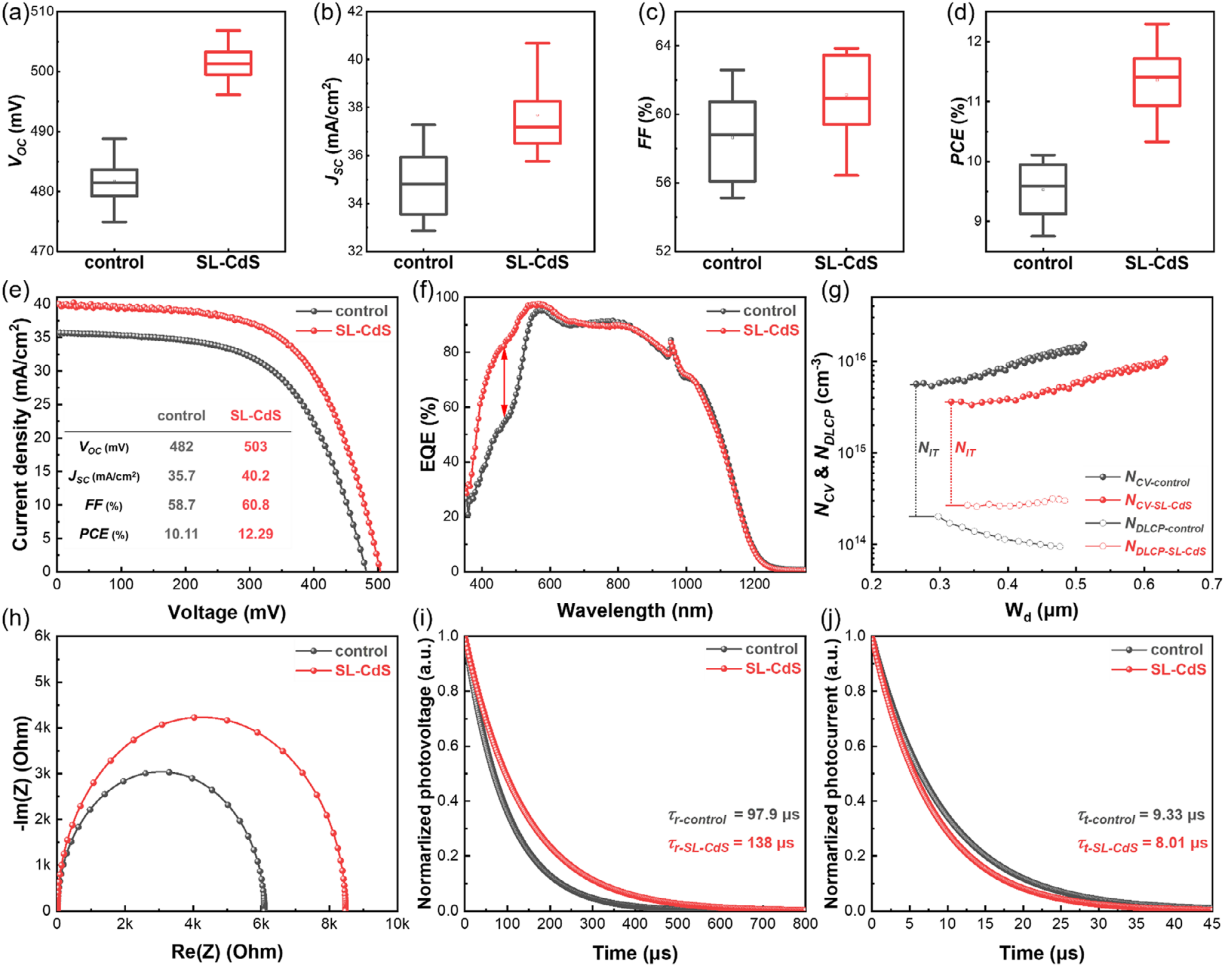
a–d) Plots of the statistical distribution of *V_OC_* (a), *J_SC_* (b), *FF* (c), and *PCE* (d) of control and SL‐CdS devices. e) *J*–*V* curves and the device structure of the flexible CZTSSe solar cells. f) EQE spectra; g) The *C*–*V* and the DLCP characteristics; h) The impedance spectra; i) TPV decay and j) TPC decay of devices.

Based on the above‐mentioned CdS CBD methods, we prepared flexible CZTSSe devices with a structure of Mo foil/CZTSSe/CdS/i‐ZnO/ITO/Ag (see Figure S5, Supporting Information). Variations in deposition processes yield distinct optimal buffer layer thicknesses, necessitating a systematic investigation into the thickness‐dependent effects of CdS on device performance. As shown in Figures S6 and S7 (Supporting Information), the SL‐CdS film samples exhibit higher uniformity across different thicknesses. While the control devices have the highest efficiency at the CdS thickness of 60 nm (Figure S6b–e, Supporting Information), SL‐CdS devices achieve higher overall performance with optimal efficiency at 30 nm thick CdS (Figure S7b–e, Supporting Information). The CdS deposition process affects the deposition rate and quality of the buffer layer film. As discussed above, CdS films prepared using the control method exhibit more holes and defects, resulting in low fill factors (*FF*) for control devices when the buffer layer thickness is reduced. The quasi‐epitaxial buffer layer prepared by the SL‐CdS method is denser. Consequently, even when the buffer layer thickness is reduced, the devices maintain a high *FF*. This enables the realization of the concept of enhancing short‐wavelength light transmittance by decreasing the CdS buffer layer thickness, significantly boosting the devices short‐circuit current. To further investigate the effects of the CBD methods on the performance of CZTSSe solar cells, we prepared 18 cells with the optimal CdS thicknesses (60 nm for control, 30 nm for SL‐CdS). As shown in **Figure** **3**a–d, the average *V_OC_* and short‐circuit current density (*J_SC_*) of SL‐CdS devices are significantly better than those of control devices. Figure 3e illustrates the *J*–*V* curves of the champion solar cells. In comparison to the control device, which has a *J_SC_* of 35.7 mA cm^−2^ and a *V_OC_* of 482 mV, the SL‐CdS device has a significantly improved *J_SC_* of 40.2 mA cm^−2^ and a *V_OC_* of 503 mV. The optimization of *J_SC_* and *V_OC_* elevates the *PCE* from 10.1% to 12.3%. The external quantum efficiency (EQE) of the SL‐CdS device is generally higher than that of the control device in the 400–600 nm range, with a maximum difference exceeding 20% (shown in Figure 3f). Due to the high quality of CdS thin films prepared by the quasi‐epitaxial strategy, a thinner thickness (about half is enough to construct a heterojunction) significantly improves the light absorption of the device.

The interfacial defect states of the devices are investigated by capacitance‐voltage (*C*–*V*) and drive‐level capacitance profiling (DLCP) characteristics, as shown in Figure 3g. The SL‐CdS device exhibits a 50.8% reduction in interface defect density (*N_IT_*) from 5.43 × 10^15^ cm^−3^ to 2.67 × 10^15^ cm^−3^, along with enhanced depletion region width and built‐in voltage compared to the control. These results demonstrate the quasi‐epitaxial heterojunction's optimization effectiveness, significantly suppressing carrier recombination via reduced interface recombination centers. Electrochemical impedance spectroscopy (EIS) is a common characterization technique for detecting carrier recombination situations in thin‐film devices. The intersection points of the Nyquist diagrams with the *x*‐axis are the recombination resistances of charges (*R_rec_*). Figure 3h shows that the SL‐CdS device exhibits a higher *R_rec_* (8.5 kΩ) compared to the control device (6.1 kΩ), indicating more effective suppression of charge transport recombination. As shown in Figure S8 (Supporting Information), both samples' *R_rec_* values decrease with increasing bias voltage. Notably, the SL‐CdS device consistently maintains higher *R_rec_* values than the control device across all the bias conditions, demonstrating effective suppression of carrier recombination throughout the space charge region and at the interface. The charge transfer and recombination characteristics of devices are further investigated by transient photovoltaic (TPV) and transient photocurrent (TPC) measurements. As shown in Figure 3i, the charge recombination lifetime (*τ_r_*) of the SL‐CdS device (138 µs) is 1.4 times that of the control device (97.9 µs) fitted from TPV curves. The longer *τ_r_* means the better suppression of recombination. The TPC decays of the devices are shown in Figure 3j. The charge transport lifetime (*τ_t_*) values of the control and SL‐CdS devices are 9.33 and 8.01 µs, respectively. A shorter *τ_t_* indicates that the SL‐CdS device has better charge transport capability. It can be concluded that the quasi‐epitaxial structure achieved through the SL‐CdS approach has significantly enhanced the quality of the CZTSSe/CdS heterojunction, effectively suppressing the recombination and improving charge transport capability in the devices.

**Figure 4:**
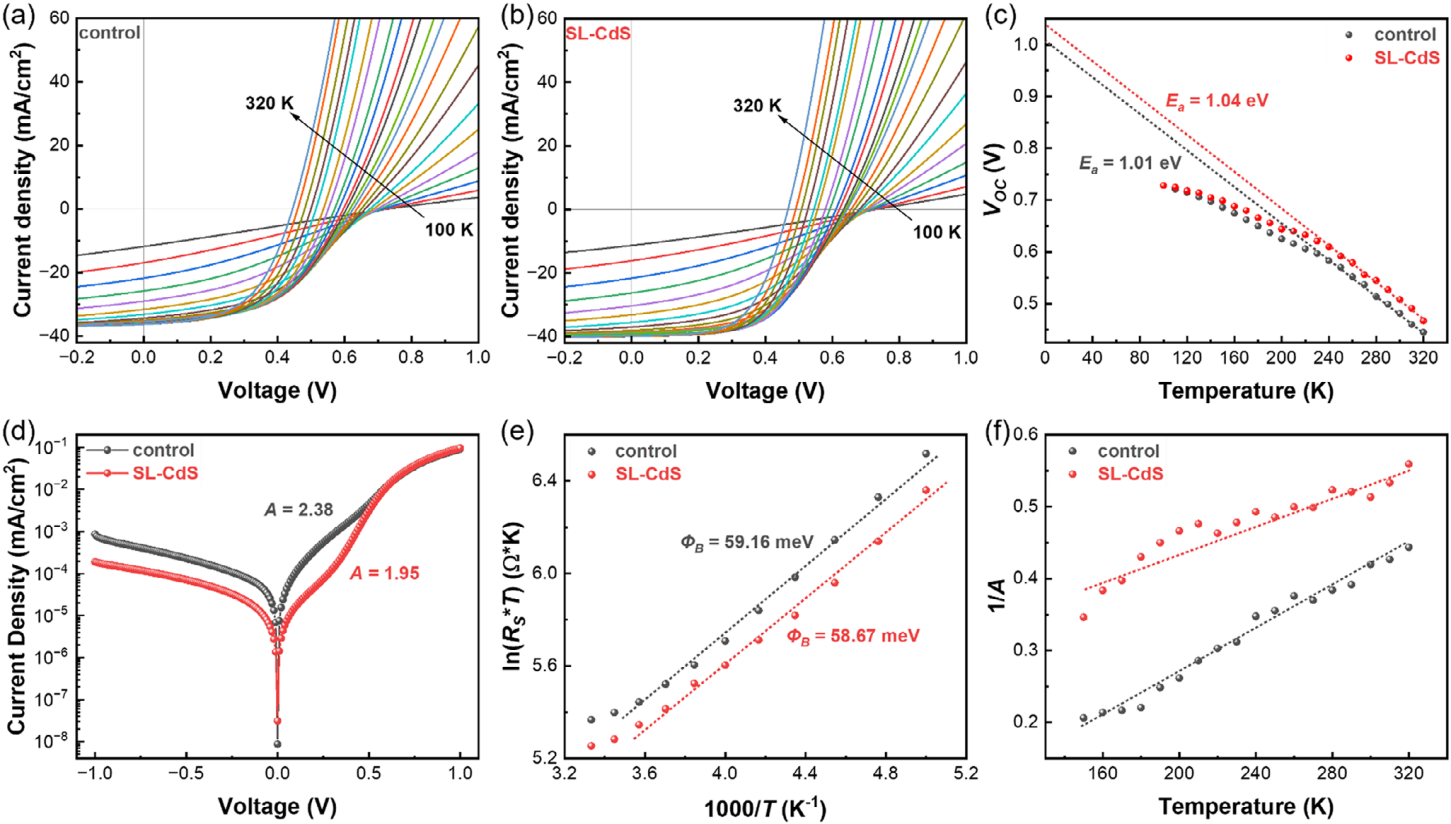
a,b) The light *J*–*V* curves of control (a) and SL‐CdS (b) devices in the temperature range of 100–320 K. c) The plots of temperature‐dependent *V_OC_* of devices. d) The dark *J*–*V* curves of CZTSSe solar cells at 300 K. e) The plots of ln(*R_S_T*) vs 1000/*T*; f) The plots of 1/*A* vs temperature of CZTSSe devices.

The carrier loss mechanism of CZTSSe devices is determined through temperature‐dependent *J*–*V* tests (see **Figure** **4**a,b). The temperature‐dependent *J*–*V* curves of the control device show lower *J_SC_*, *FF*, and *V_OC_* than those of the SL‐CdS device above 150 K. Below 150 K, the *J*–*V* curves of the two devices tend to converge, with *V_OC_* increasing and *J_SC_* decreasing. This is attributed to the influence of low temperatures on defects and intrinsic excitation. The analysis of the diode activation energy (*E_a_*) is calculated through *T*‐*V_OC_* measurements as follows:^[^ ^44^ ^]^

(5) $$V_{\mathrm{OC}}=\frac{E_{a}}{q}-\frac{\mathrm{AkT}}{q}\ln\left(\frac{J_{00}}{J_{\mathrm{SC}}}\right)$$

where *q* is the elementary charge, *k* is the Boltzmann constant, *T* is the temperature, *A* is the diode ideality factor, *J_00_* is a current pre‐factor with weak temperature dependence. The *E_a_* can be determined by the intersection between the extended linear segment of the *V_OC_*‐*T* curve with the *y*‐axis. As shown in Figure 4c, the *E_a_* of the SL‐CdS device is 1.04 eV, higher than that of the control device (1.01 eV) and closer to the *E_g_* of CZTSSe. This indicates that interface recombination in the SL‐CdS device is more effectively suppressed.

The *A* and series resistance of the diode, calculated from the dark *J*–*V* curve, are used to assess the quality of the PN junctions and recombination behaviors in solar cells. As shown in Figure 4d, at room temperature, the *A* value of the SL‐CdS device is 1.95, significantly lower than the 2.38 of the control device. A smaller *A* value indicates better PN junction quality and fewer carrier recombination events. To elucidate carrier recombination mechanisms, we performed temperature‐dependent dark *J*–*V* characterization to quantify two critical loss pathways: back‐contact potential barrier and heterojunction interface recombination. The back barrier height (*Φ_B_*) of devices can be determined from the dark *J*–*V* curves as the following equation:^[^ ^44^ ^]^

(6) $$R_{S}=\frac{k}{qR^{*}T}\exp\left(\frac{\Phi_{B}}{\mathrm{kT}}\right)$$

where *R_S_* is the series resistance at different temperatures, R^*^ is the effective Richardson constant. By calculating the slope of the fit of ln (*R_S_T*) vs 1000/*T* curves, we can obtain the *Φ_B_* values of the devices. As can be seen from Figure 4e, the *Φ_B_* of the SL‐CdS device differs from that of the control device by only 0.49 meV, indicating that the difference in charge transport capability of the two devices does not originate from the back potential barrier. The change in the *A* with temperature is caused by tunnel‐enhanced interface recombination, as shown in the following equation:^[^ ^44^ ^]^

(7) $$A=\frac{1}{\alpha }\frac{E_{00}}{kT}\coth\left(\frac{E_{00}}{kT}\right)$$

where α is the ratio of the potential barrier to the flat band height, and *E_00_* is the characteristic tunneling energy. Generally, the value of *E_00_* is much smaller than *kT*, so the formula (3) can be simplified as follows:

(8) $$\frac{1}{A}=\frac{\alpha }{E_{00}}kT$$

*E_00_/α* can be obtained from the slope of 1/*A‐T*. Since α is difficult to determine, we will treat *E_00_/α* as the equivalent tunneling energy of the device under test. As shown in Figure 4f, the *A* value of the CZTSSe device increases with decreasing temperature and exhibits a linear trend, indicating the presence of tunnel‐enhanced interface recombination in the devices. Tunnel‐enhanced interface recombination enhances nonradiative recombination within devices, thereby reducing*V_OC_* and *FF*. The interface defect states can act as “midway stations” for tunneling, enabling electrons and holes to directly recombine via quantum tunneling. This significantly enhances tunnel‐enhanced interface recombination. The *E_00_/α* of the SL‐CdS device is 1.52 times that of the control device (88.3 to 58.2 meV). The substantial elevation in tunneling energy signifies a raised carrier tunneling barrier at the interface, which will inevitably suppress tunnel‐enhanced recombination. These experimental results further validate the effective mitigation of carrier recombination in CZTSSe devices through quasi‐epitaxial heterojunction engineering.

**Figure 5:**
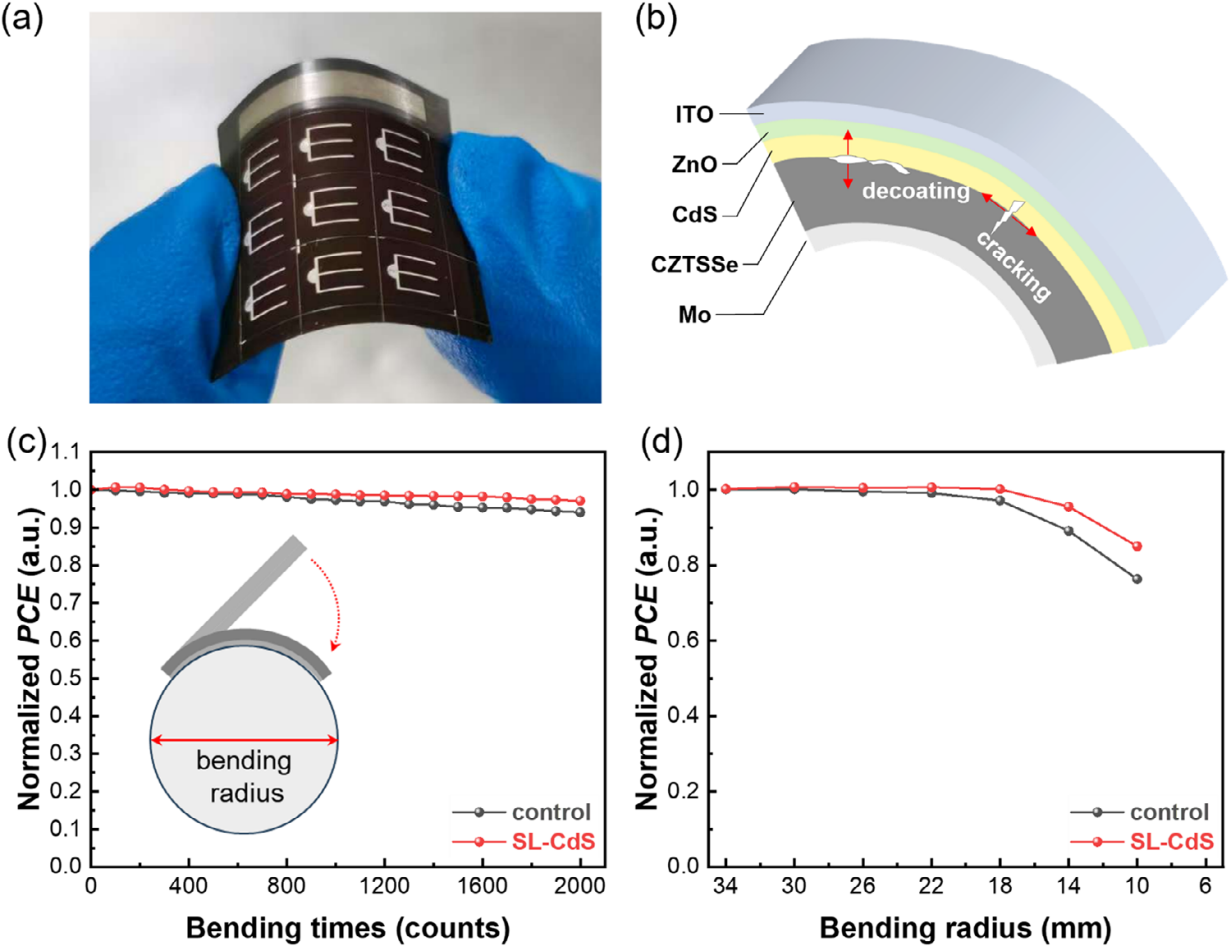
a) The photograph of a bending flexible CZTSSe solar cell. b) Schematic diagram of internal damage to flexible CZTSSe solar cells during bending. c,d) The efficiency degradation curves with bending times (c) and bending radius (d).

**Figure** **5**a presents the bending state of a flexible CZTSSe solar cell fabricated on a 50 nm‐thick Mo foil. The bending‐induced efficiency degradation serves as a critical performance metric for flexible solar cells. As illustrated in Figure 5b, the performance deterioration primarily stems from internal damage and microcracking in the thin films caused by accumulated stress and mechanical bending forces. Consequently, quantitative analysis of efficiency decay provides a reliable method for assessing the stress relief characteristics of thin films in flexible solar devices. The flexible CZTSSe device is bent around a cylinder with a fixed radius, which is defined as the bending radius of the device, and the average *PCE* of all the cells is measured after every 100 bending cycles. Figure 5c shows the effect of cyclic bending times on *PCE* at a radius of 22 mm. Flexible CZTSSe solar cells prepared by both the control and the SL‐CdS methods demonstrated excellent bending tolerance, with less than 10% *PCE* reduction after 2000 bending cycles. But it can be observed that the *PCE* of the SL‐CdS device remains higher than that of the control device after multiple bending cycles. To further compare the differences in bending tolerance between the two sample devices, the *PCE* degradation curves at different bending radius are characterized, as shown in Figure 5d. When the radius is less than 18 mm, the device *PCE* decreases significantly, indicating that cracking damage within the thin films significantly restricts carrier transport. However, the *PCE* of the SL‐CdS device after degradation remains higher than that of the control device, indicating that it suffers less internal damage under extreme bending conditions. Within the CdS thin film, large‐sized particles and pores generate severe internal stress, which can cause significant damage after bending. The above results indicate that the quasi‐epitaxial CdS layer simultaneously achieves effective suppression of interface defects in the CZTSSe/CdS heterojunction and significant enhancement of mechanical robustness in flexible CZTSSe photovoltaic devices.

## 3 Conclusion

In summary, the quasi‐epitaxial heterojunction of flexible CZTSSe solar cells is constructed through ammonia atmosphere control and parameter optimization in the CBD process. The sealed NH_3_ environment enhances the stability of complex molecules and reduces molecular kinetic energy, thereby strictly controlling the formation rate and size of CdS molecular clusters. The slow and dense deposition of molecular clusters onto the CZTSSe film simultaneously suppresses interfacial element diffusion and facilitates quasi‐epitaxial growth of hexagonal CdS. Consequently, heterojunction interface defect density is decreased by 51%, while tunnel‐enhanced recombination is significantly suppressed, collectively enhancing *V_OC_*. Epitaxial growth enables a thinner CdS buffer layer with reduced bulk defect density. This simultaneously enhances short‐wavelength transmittance in the window layer, boosting *J_SC_* by 15%, and significantly improves charge carrier separation and transport within the device. Consequently, the optimized flexible CZTSSe solar cells achieve an efficiency of 12.3% with a *V_OC_* of 503 mV, a *J_SC_* of 40.2 mA cm^−2^, and an *FF* of 60.8%. Furthermore, the quasi‐epitaxially grown CdS film mitigates performance degradation in flexible CZTSSe devices under bending stress. This CBD quasi‐epitaxial growth strategy thus presents a promising approach for advancing flexible kesterite solar cells.

## Conflict of Interest

The authors declare no conflict of interest.

## Supporting information



Supporting Information

## Data Availability

The data that support the findings of this study are available from the corresponding author upon reasonable request.
