# Supplementary material for: Molecular Cluster‐Controlled Quasi‐Epitaxial CZTSSe/CdS Heterojunction Enables 12.3% Efficiency of Flexible Solar Cells
Source: Adv Sci (Weinh). 2025 Dec 8;13(9):e20208. doi: 10.1002/advs.202520208 (PMC12903968; doi:10.1002/advs.202520208)
Supplement: Supplementary file 1 — Supporting Information [file ADVS-13-e20208-s001.docx]

**Supporting Information**

**Molecular Cluster-controlled Quasi-epitaxial CZTSSe/CdS Heterojunction Enables 12.3% Efficiency of Flexible Solar Cells**

Weihao Xie^1^, Yifan Li^1^, Quanzhen Sun^1^, Weihuang Wang^1^, Renjie Wang^1^, Caixia Zhang^1,2^, Jionghua Wu^1^, Hui Deng^*,1^, Shuying Cheng^*,1,2^

^1^College of Physics and Information Engineering, Institute of Micro-Nano Devices and Solar Cells, Fuzhou University, Fuzhou, 350108, P. R. China.

^2^Jiangsu Collaborative Innovation Center of Photovoltaic Science and Engineering, Changzhou 213164, P. R. China.

*Corresponding author email: sycheng@fzu.edu.cn (S. Cheng); denghui@fzu.edu.cn (H. Deng).

**Experimental section**

**Deposition of CZTSSe films**

CuCl_2_ (0.38 mmol), Zn(CH_3_CO_2_)_2_ (3 mmol), SnCl_4_ (2.5mmol), CH_4_N_2_S (0.02 mol), AgCl (0.2 mmol), and LiCH_3_CO_2_ (0.4 mmol) were dissolved in 2-Methoxyethanol (C_3_H_8_O_2_, 10 mL), and then kept stirring for 120 min at 60℃ to obtain a clear solution. The CZTSSe solution was spin-coated on the Mo foil and annealed in Ar_2_ at 320°C for 2 min to fabricate CZTSSe precursor films. The precursor films and solid selenium particles were rapidly annealed in a graphite box of a tube furnace at 560℃ for 780 seconds, and the cavity was filled with nitrogen. During the entire annealing process, a pressure of 10^5^ Pa was maintained in the chamber by flowing nitrogen. After the annealing completed, the samples were cooled naturally in the RTP chamber.

**Deposition of quasi epitaxial CdS films**

The CdS film was prepared by optimized chemical bath deposition. In a beaker, 32 mL CdSO_4_ solution (0.015 mol/L) was mixed with 40 mL NH_3_∙H_2_O and 220 mL deionized water. After stirring for 5 min at 70°C, we added 16 mL of thiourea aqueous solution with a concentration of 1.5 mol/L. Then we placed the sample in the solution and deposited the CdS film. The CBD process conducted in the open beaker and sealed beaker (using plastic wrap and rubber bands) is designated as “control” and “S-CdS”, respectively. A solution of CdSO₄ was mixed with ammonia water and deionized water, then heated in water at 60°C for 2 minutes (without stirring). The sample was then placed in the solution with added thiourea solution, and the beaker was sealed. Stirring was initiated once the solution turned yellow. This process is referred as “SL-CdS.”

**Fabrication of solar cells**

Mo foils were electro-polished at a current of 150 mA in a mixed solution of methanol and sulfuric acid, followed by drying to get smooth and clean surfaces. The CZTSSe solar cells were fabricated in the structure of Mo foil/CZTSSe/CdS/ZnO/ITO/Ag with an MgF_2_ antireflection layer. The thicknesses of the Mo substrate, ZnO, ITO, Ag, and MgF_2_ films were 50 μm, 50 nm, 200 nm, 500 nm, and 80 nm, respectively. ZnO and ITO films were deposited using low-temperature RF magnetron sputtering. The Ag electrode and MgF_2_ anti-reflection layer were subsequently deposited via thermal evaporation. Each sample was mechanically scribed into nine tiny cells, each having an effective area of 0.205 cm².

**Characterizations and measurements**

The transmission spectra were measured using a UV–visible spectrophotometer (Agilent CARY 5000 Scan). The cross-section test samples were prepared using the focused ion beam (FIB) technique (Helios G4 CX). High-resolution transmission electron microscopy (HRTEM) and energy dispersive spectroscopy (EDS) were carried out by a 200 kV field emission transmission electron microscope (Talos F200X G2, Thermofisher Scientific). The *J-V* data of the devices were collected by a Keithley 2400 source meter when the device was under standard simulated 1 sun illumination (AM1.5G, 100 mW/cm^2^) with a sweep range from -0.1 to 0.52 V. The external quantum efficiency (EQE) spectra of the devices were measured by a EQE test system (CT-SC-T QE, CROWNTECH). The temperature-dependent *J-V* characterizations of the devices were characterized by a semiconductor characterization system (Fs-Pro, Hong Kong) with the substrate temperature range from 100 to 320 K at an interval of 10 K. The space charge limited current (SCLC) characterizations were performed by a semiconductor characterization system (Fs-Pro, Hong Kong). The capacity-voltage (*C-V*) and drive-level capacity (DLCP) characterizations of the devices were conducted by Keithley 4200 under a frequency of 50 kHz. The electrochemical impedance spectroscopy (EIS) of the devices was carried out in the dark by an electrochemical workstation (Bio-Logic SAS, VPS). The Fluorescence Spectroscopy (FL) was characterized via an FLS1000 spectrograph.

**Figures and captions**


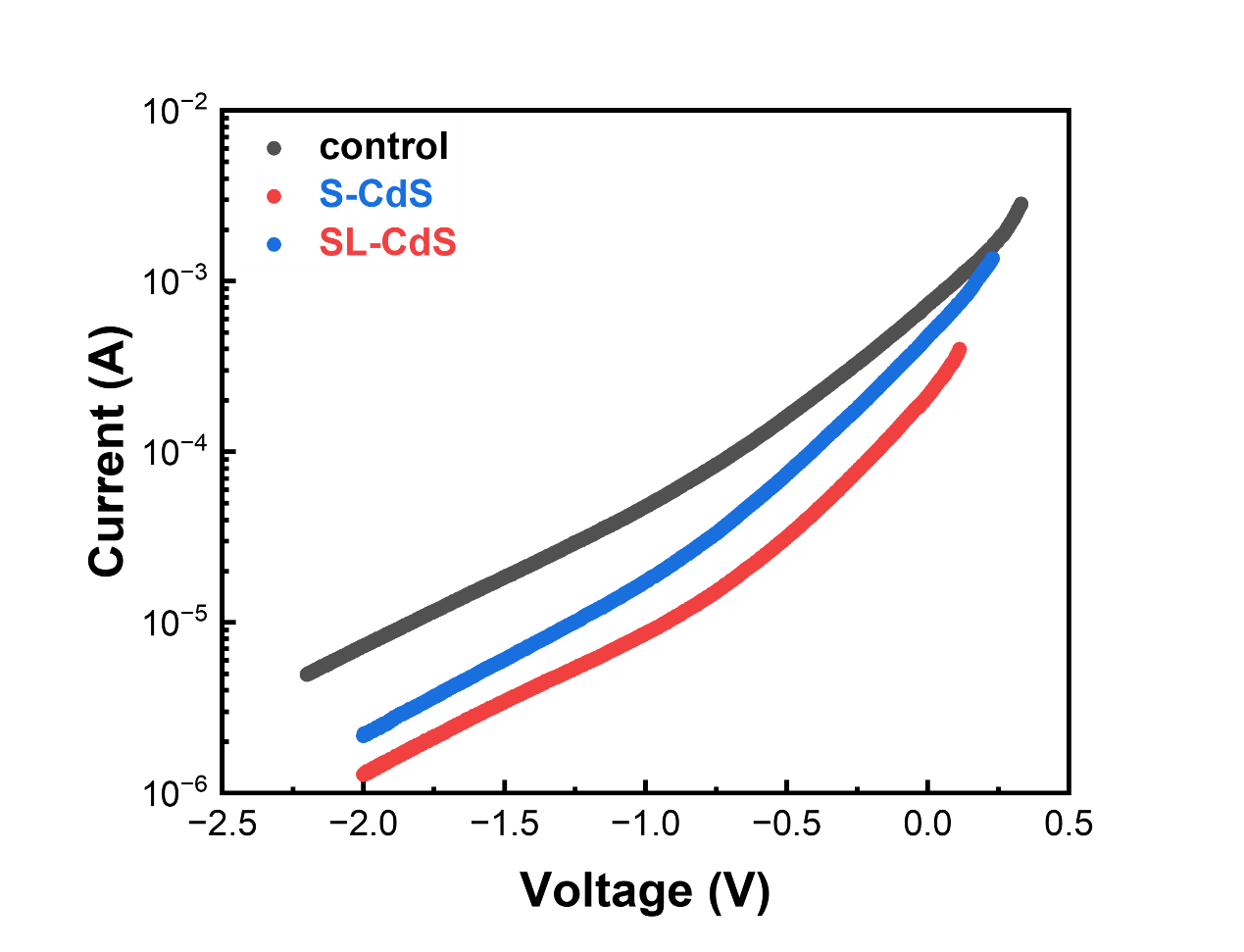


**Figure S1.** SCLC curves of CdS thin films prepared by control, S-CdS and SL-CdS methods, respectively.

The space charge limited current (SCLC) of the CdS films is measured to directly characterize the defects densities. For characterization, we fabricated the samples with ITO/CdS/Ag structure. When the electric field is small, there is a proportional linear relationship between the current and voltage of the CZTSSe film. As the electric field increases, the defect states in the space charge region are filled, and the current increases significantly. When the trap is filled, the relationship between current and voltage conforms to the Mott-Gurney law:

$J=\frac{9\varepsilon_{0}\varepsilon_{r}\mu V^{2}}{8L^{3}}$ (SI-1)

Where *μ* is the carrier mobility, *V* is the measured material volume and *L* is the electrode distance. When the voltage reaches the filling limit voltage (*V_TFL_*), the increase of the current changes from linear to quadratic. Meanwhile, the defect density (*N_t_*) of the film and *V_TFL_* conform to the following formula:

$V_{TFL}=\frac{qN_{t}L^{2}}{{2\varepsilon}_{0}\varepsilon_{r}}$ (SI-2)

The logarithm of *I-V* curves is adopted to find the *V_TFL_* conveniently. The *V_TFL_* values for control sample, S-CdS sample, and SL-CdS sample are 1.107 V, 0.615 V, and 0.265 V, respectively. According to formula (SI-2), the *N_t_* values of control sample, S-CdS sample, and SL-CdS sample are 2.10×10^17^ cm^-3^, 1.17×10^17^ cm^-3^, and 5.03×10^16^ cm^-3^.


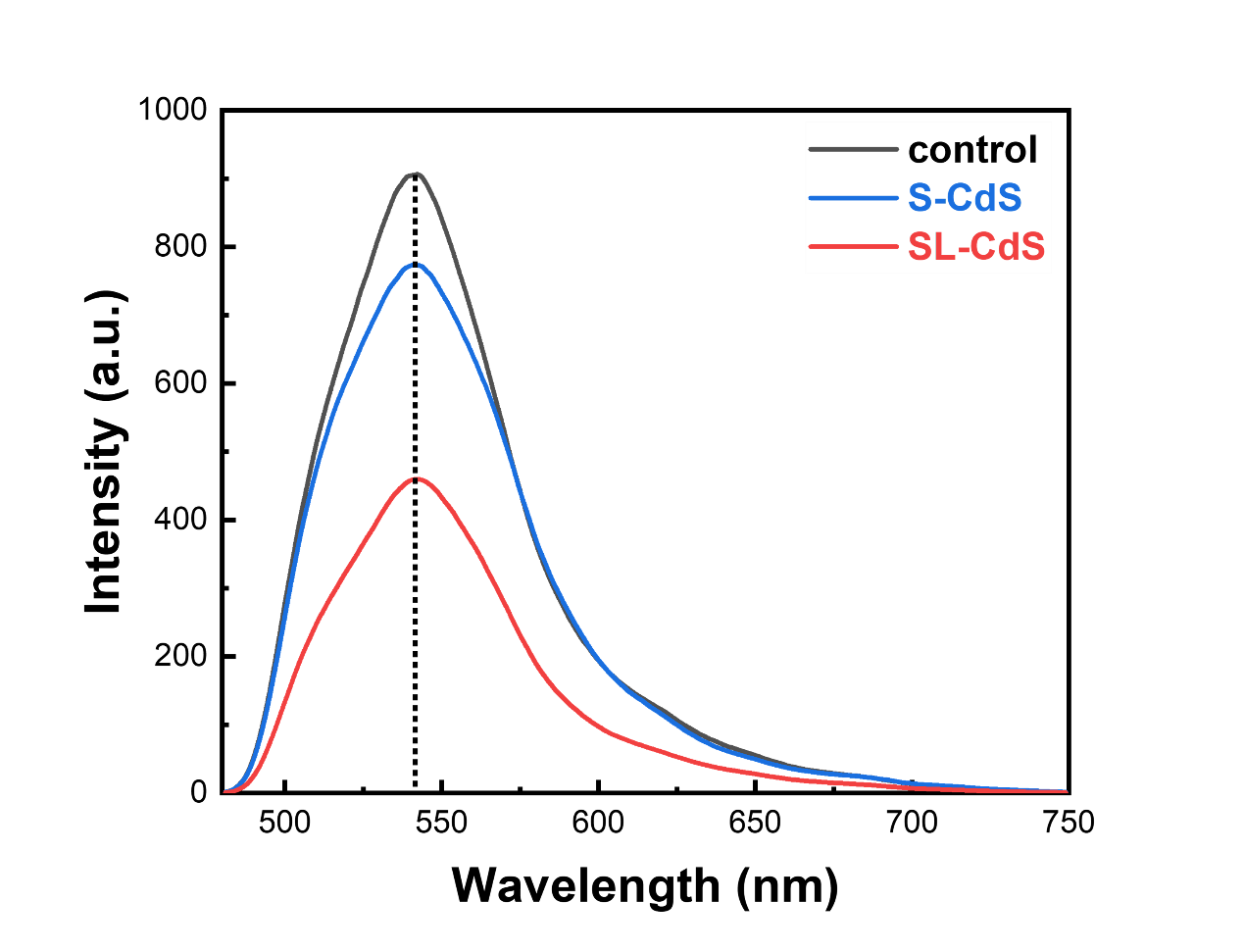


**Figure S2.** FL spectra of ITO/CdS samples prepared by control, S-CdS and SL-CdS methods, rspectively.


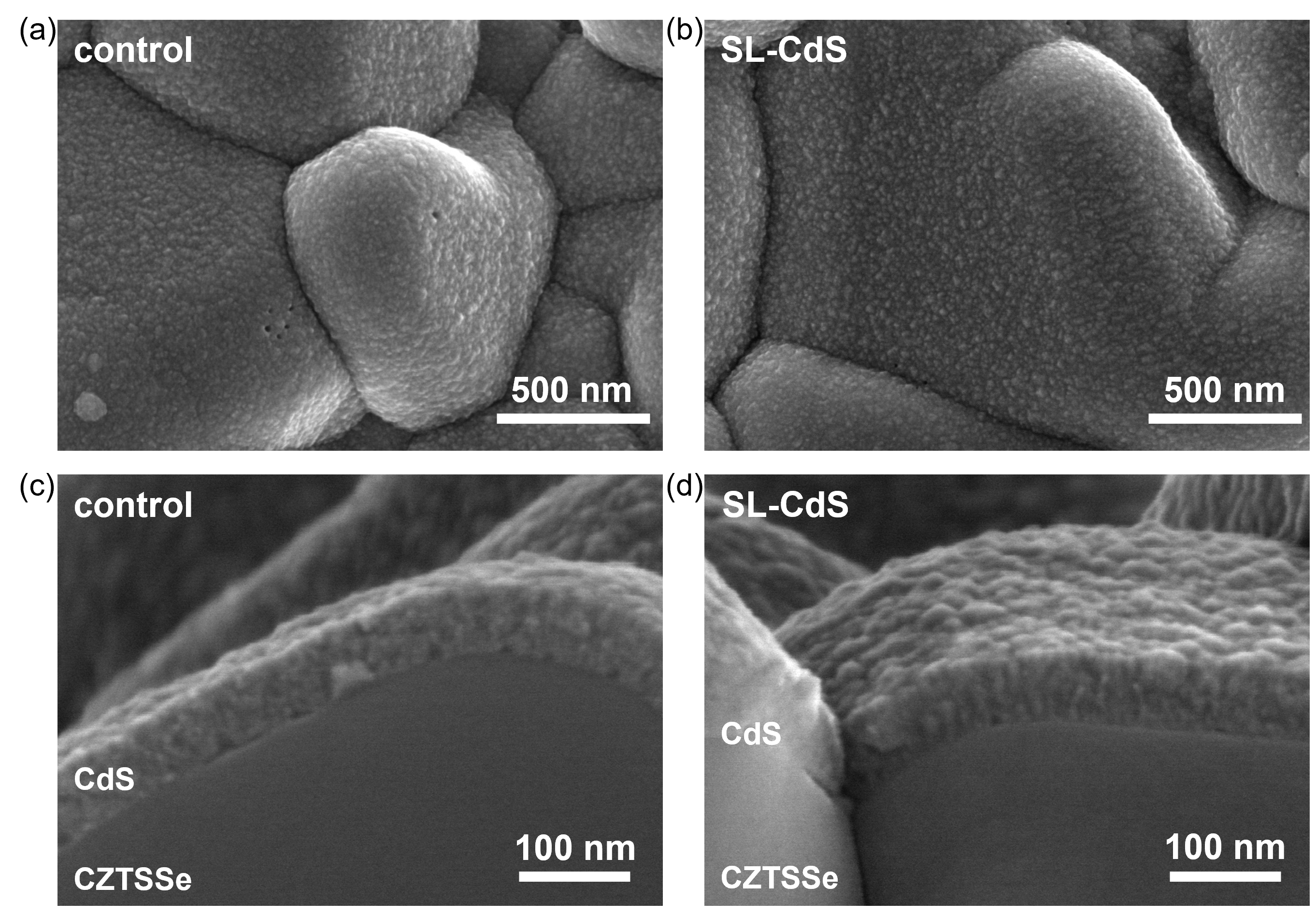


**Figure S3**. a-b) Top-view SEM images of the CZTSSe/CdS heterojunction prepared by control method (a) and SL-CdS method (b). c-d) Cross-section SEM images of the CZTSSe/CdS heterojunction prepared by control method (c) and SL-CdS method (d).


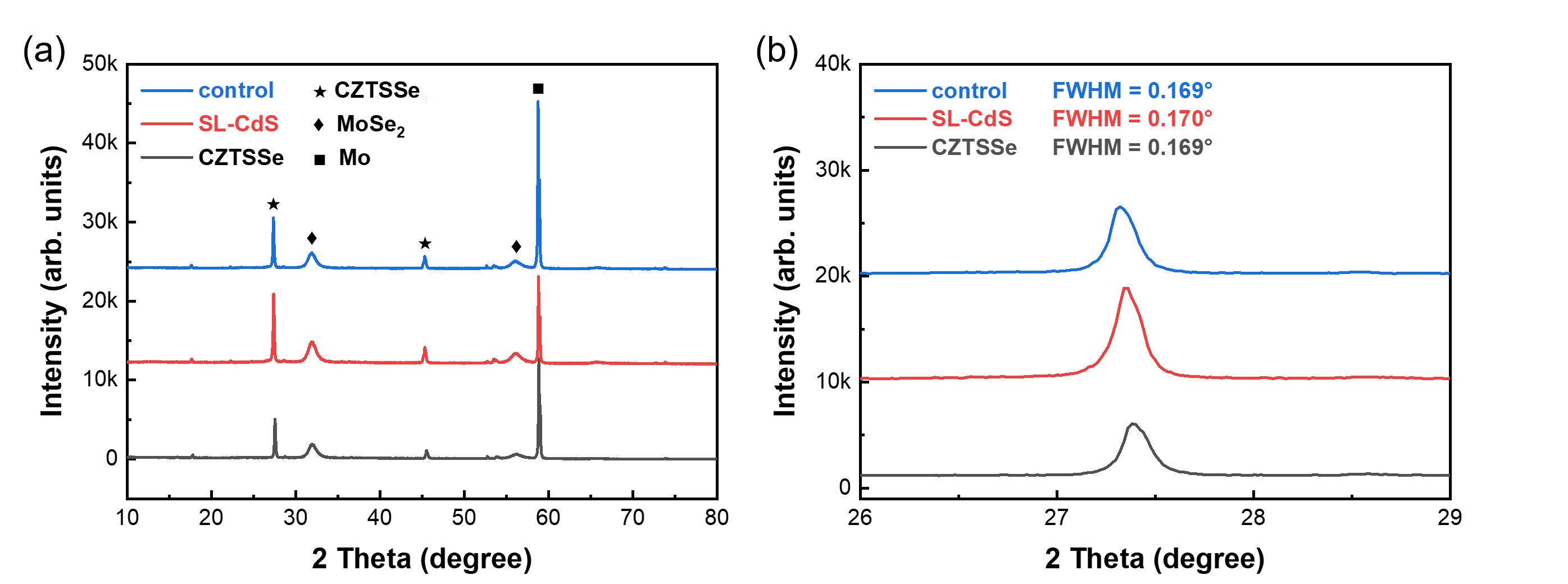


**Figure S4.** a)XRD patterns and b) local magnification plots of (112) peaks of the CZTSSe/CdS samples.


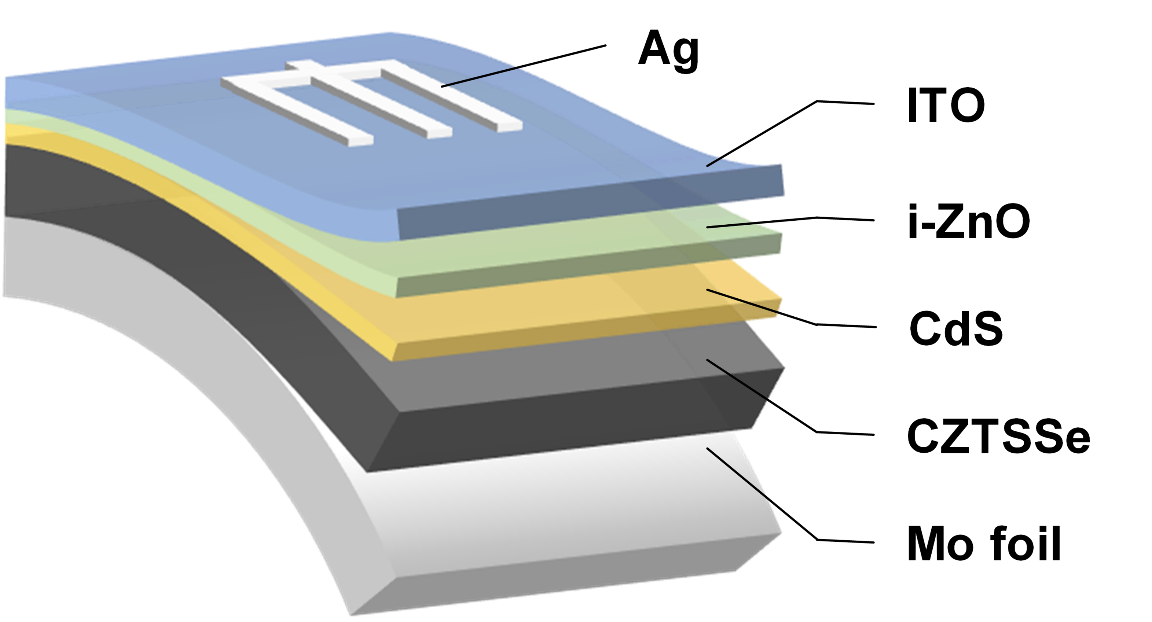


**Figure S5.** The device structure of the flexible CZTSSe solar cell.


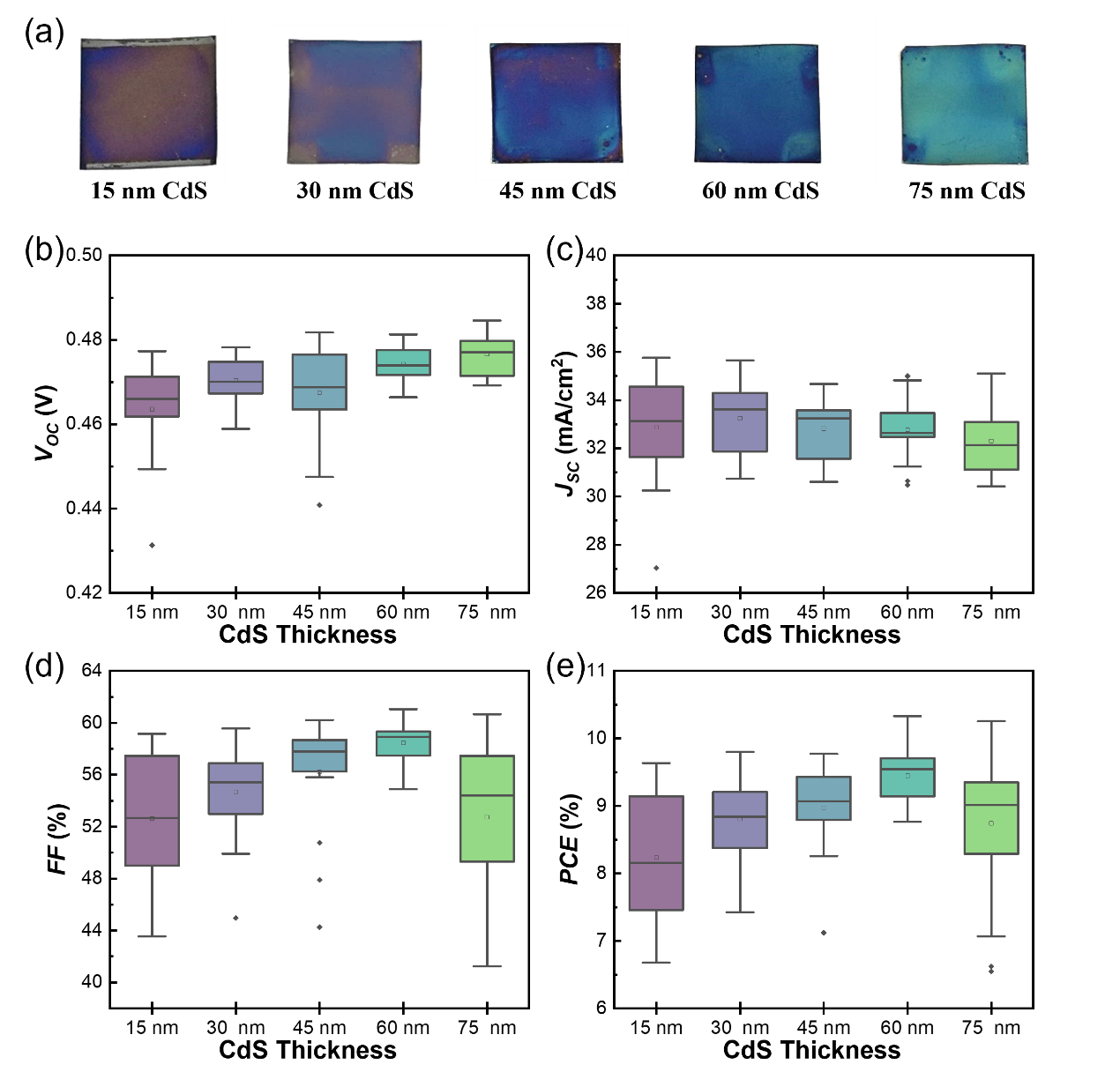


**Figure S6.** (a) Photographs of CdS films with varied thicknesses prepared by the control method on CZTSSe films. And plots of the statistical distribution of (b) *PCE*, (c) *V_OC_*, (d )*J_SC_*, and (e) *FF* from different CdS thicknesses for devices prepared by control mehod.


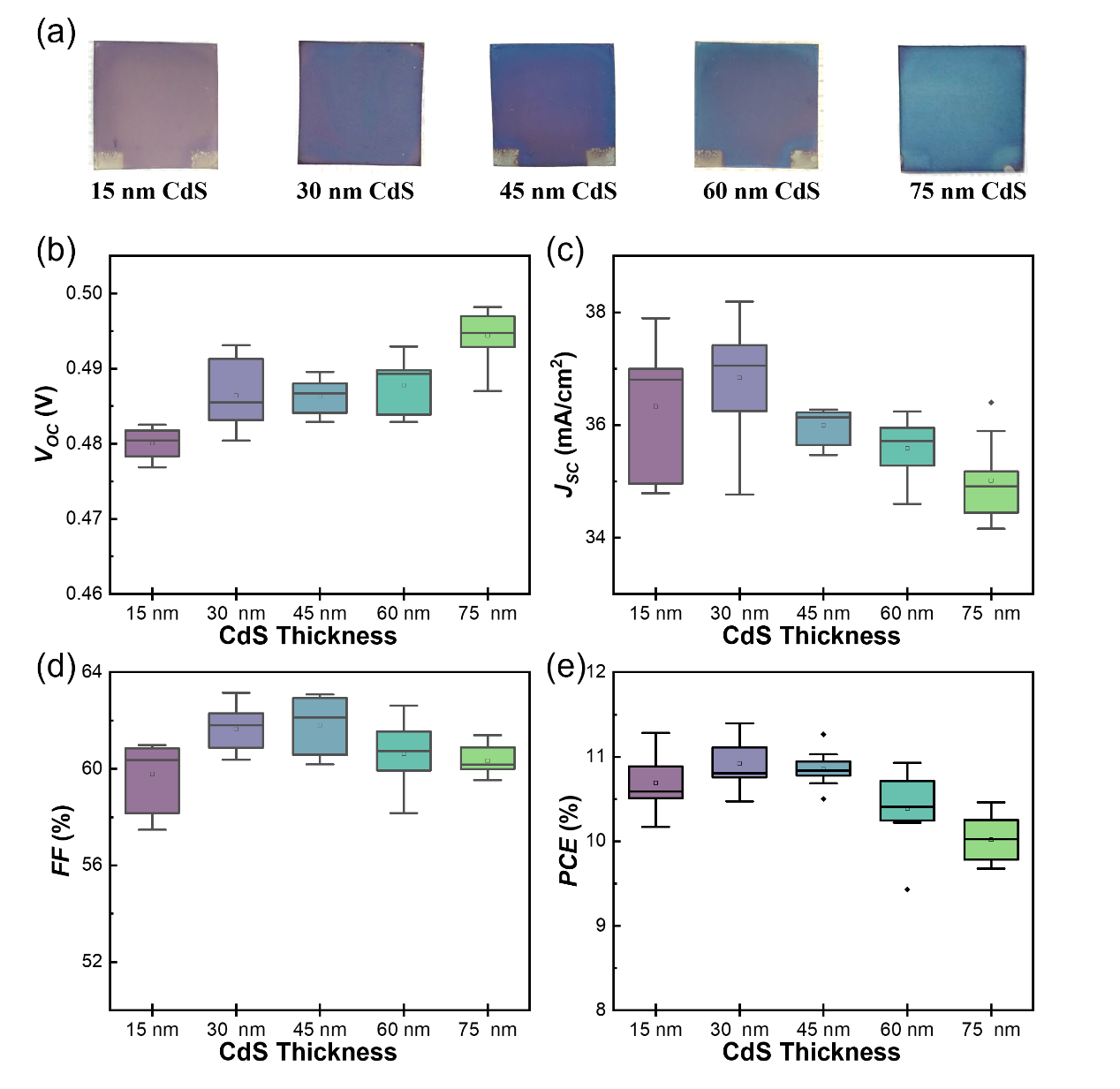


**Figure S7.** (a) Photographs of CdS films with varied thicknesses prepared by the SL-CdS method on CZTSSe films. And plots of the statistical distribution of (b) *PCE*, (c) *V_OC_*, (d) *J_SC_*, and (e) *FF* from different CdS thicknesses for devices prepared by SL-CdS method.


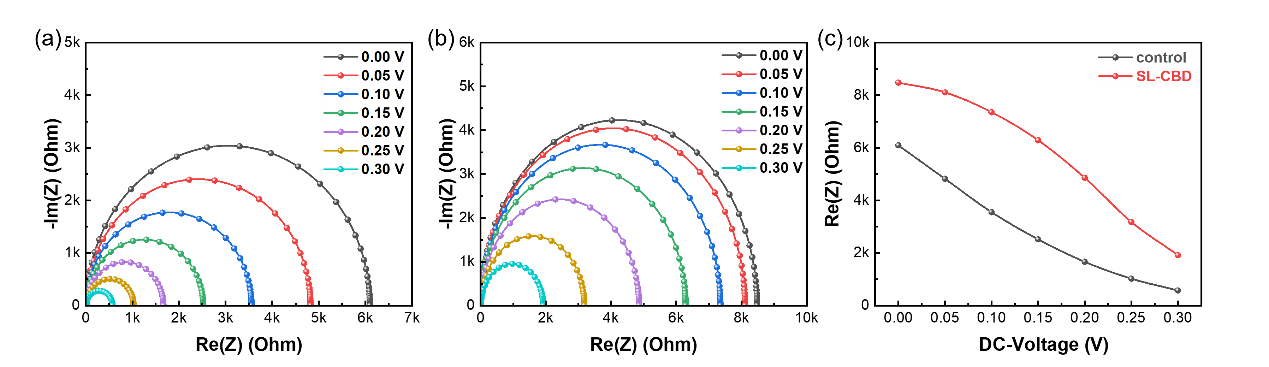


**Figure S8.** Bias voltage-dependent impedance spectra of (a) control device and (b) SL-CdS device. (c) bias voltage-dependent recombination resistance curves for control and SL-CdS devices.
